# Recent Medico-Legal Decisions

**Published:** 1889-11

**Authors:** 


### Selections.

---

### RECENT MEDICO-LEGAL DECISIONS.

MARSHALL D. EWELL, M. D., Attorney-at-law, in the *North American Practitioner*, notes the following cases that have been published since his last communication :

### DRUGGISTS.

If the owners of a drug-store take any part in conducting the business, and are not licensed as required by N. H. Gen. Laws, Chap. 133, they are liable to a penalty, although they have in their employ a person duly licensed, who compounds the medicines called for by physicians' prescriptions. *State v. Forcier* (N. H.), 17 Atl., 577.

A fee of five dollars for a license to engage in the business of an apothecary and druggist is merely an equivalent for the services in making the examination and issuing the license, and cannot be considered as a tax upon the business, or of depriving the applicant of his property without due process of law. *Id.*

It is no defense to a prosecution for retailing drugs, etc., without license, to show that no actual harm has resulted from defendant's violation of the statute. *Id.*

Under the Tennessee statute, no druggist, simply because he uses alcoholic or vinous liquors in the compounding of tinctures, essences, or other preparations, is therefore subject to the tax of a liquor-dealer. *The Druggist Cases*, 85 Tenn., 449.

Druggists selling liquors contrary to the Tennessee Acts of 1870 and 1885, and without license, are subject not only to the tax imposed on liquor-dealers, but also to indictment for each sale. *Id.*

Under Tennessee Act 1885, Chap. 5, it is not lawful for a druggist to sell, without paying the tax on liquor-dealers, either spirituous or vinous liquors for any purpose whatever, upon prescription or otherwise, " except wine for sacramental purposes." *Id.*

Prior to Tennessee Act 1885, Chap. 5, a druggist might, in good faith, sell wine for communion purposes, or fill the prescription of a regular practicing physician for either alcoholic or vinous liquors, without paying the tax on liquor-dealers, but at no time since Tennessee Act 1870, Chap. 51, has it been lawful for him to sell for medical purposes, without such prescription. *Id.*

Under the Tennessee statutes, no druggist selling compounds, tinctures, essences, perfumery, or other preparations of which either alcohol, wine, or other liquor is a component part, subjects himself to the tax imposed on liquor-dealers, unless such sale is a mere sham or subterfuge to evade the law. *The Druggist Case*, 85 Tenn., 449.

### INSANITY.

Where there is evidence tending to prove that defendant, at the time of committing a homicide, was in fact insane as distinguished from mere intoxication, an instruction that if his mental condition was directly, or even remotely caused by voluntarily drinking intoxicating liquors, or produced by other causes combined with such voluntary drinking, he must be considered responsible and sane, is misleading. *Terrill v. State* (Wis.), 42 N. W., 243.

The fact that a grantor, because of financial troubles, is in a condition of mind to make a foolish bargain, is of no weight to prove him insane, so as to invalidate a conveyance of land by him. *De Witt v. Mattison* (Neb.), 42 N. W., 742.

Evidence that the assured was ordinarily a man of pleasant and genial disposition, whose family relations were pleasant, that after a certain time he became depressed, complained of pain in his head, was abstracted and stupid, could not pay close attention to business, did not appear to remember what was told him, and impressed people with whom he came in contact that he was out of his right mind, and that he finally cut his own throat—is sufficient to go to the jury upon the question of insanity. *Blackstone v. Standard L. & A. Ins. Co.* (Mich.), 3 L. R. A., 486; 39 Alb. L. J., 449; 42 N. W., 156.

### PRACTICE ACTS.

A statute putting upon a physician who gives a prescription for whisky the burden of showing that the whisky was needed as a medicine by the patient is not unconstitutional. *Com. v. Minor* (Ky.), 10 Ky. L. P. Rep., 1008; 11 S. W., 472.

An act prohibiting a physician from giving a written prescription for whisky, except to a person who is actually sick, and who needs such liquor as a medicine, is not unconstitutional. *Id.*

A physician cannot be convicted, under Kentucky Act, May 9, 1884, Section 8, for giving a prescription for whisky, if the person to whom it is given is actually sick, and if, after a reasonably full and fair investigation of the disease, the physician in good faith believes that the patient needs whisky as a medical remedy. *Id.*

Under the North Carolina statute, a physician prescribing liquor for a minor, and selling or giving it to him, he keeping the liquor for sale or profit, is guilty of a misdemeanor, although he may act in good faith. *State v. McBrayer*, 98 N. C., 619.

A contract to pay a fee for services rendered by a physician who is not licensed, is void in its inception, where a statute prohibits him from practising as a physician for fee or reward. *Puckett v. Alexander* (N. C.), 3 L. R. A., 43; 17 Wash. L. Rep., 311; 8 S. E., 767.

An amendment to a statute prohibiting a person from practising medicine without a license, which provides that it shall not apply to physicians who have a diploma from a regular medical college, cannot make valid a contract made before the passing of the amendment to pay for the services of an unlicensed physician who had such a diploma, which contract was void in its inception. *Id.*

Services rendered by an unlicensed physician, under a contract which was void in its inception because prohibited by statute, do not constitute a consideration which will support an express promise to pay for the services. *Puckett v. Alexander* (N. C.), 3 L. R. A., 43; 17 Wash. L. Rep., 311; 8 S. E., 767.

---

L. C. CARR, M. D., Professor of Obstetrics, Cincinnati College of Medicine and Surgery, Cincinnati, O., says: "I have given 'Papine' (Battle) a fair trial, and am well pleased with its action, especially so in the case of an infant suffering with an attack of convulsions. Its action was speedy and safe."

